# The *Mycobacterium tuberculosis* H37Ra gene MRA_1916 causes growth defects upon down-regulation

**DOI:** 10.1038/srep16131

**Published:** 2015-11-04

**Authors:** Kumar Sachin Singh, Sudheer Kumar Singh

**Affiliations:** 1 Microbiology Division, CSIR-Central Drug Research Institute, B.S. 10/1, Sector 10, Jankipuram Extension, Sitapur Road, Lucknow, U.P., India, Pin: 226031

## Abstract

D-amino acid oxidases play an important role in converting D-amino acids to their corresponding α-keto acids. MRA_1916 of *Mycobacterium tuberculosis* H37Ra (*Mtb*-Ra) is annotated to be a D-amino acid oxidase (DAO). However, not much information is available about its physiological role during *Mtb*-Ra growth and survival. The present study was taken-up to understand the role of DAO during different stages of growth and effect of its down-regulation on growth. Recombinant *Mtb*-Ra strains with DAO and GlcB (malate synthase: MRA_1848) gene knockdown were developed and their growth was studied using Microtiter Alamar Blue Assay (MABA) with glycerol, acetate and glycine as a carbon source. Ethyl bromopyruvate (BrP) was used as an inhibitor of GlcB. MABA study showed inhibition of wild-type (WT) and knockdowns in the presence of BrP (2.5mM). However, growth inhibition of WT was less noticeable at lower concentrations of BrP. *Mtb*-Ra with DAO knockdown showed poor utilization of glycine in the presence of BrP. The DAO localization study showed its prominent distribution in cytosolic fraction and to some extent in cell wall and membrane fractions. Growth profile of WT under oxygen and nutritional stress showed changes in expression of DAO, GlcB, PckA (phosphoenolpyruvate carboxykinase: MRA_0219) and GlyA1 (serine hydroxymethyltransferase: MRA_1104).

Tuberculosis caused by *Mycobacterium tuberculosis* (*Mtb*) remains a major cause of deaths in developing countries with subsequent increase in healthcare cost and economic burden to society. Almost one third of the world population is latently infected by this bacillus. The *Mtb* has evolved remarkable mechanisms to evade the host immune response and can remain dormant for long periods of time. Knowledge regarding the availability of nutrients for the survival of bacilli inside the macrophage remains obscure and is an area of intensive research. The earlier studies on *Mtb* metabolism observed preferential utilization of lipids by *Mtb* when isolated from the lung tissues of mice^1^. Further studies confirmed that enzymes of glyoxylate shunt were important for growth and virulence of *Mtb*. Recombinant *Mtb* lacking isocitrate lyase isoforms (*icl1* and *icl2*) failed to establish infection and got cleared from the lungs of infected mice^2^,^3^. Malate synthase (GlcB) is the second enzyme of glyoxylate shunt. It is responsible for the conversion of glyoxylate into malate and uses an acetyl-CoA during this conversion. The malate formed by GlcB activity again enters TCA cycle. Enzymes of glyoxylate pathway including isocitrate lyase isoforms and malate synthase play an important role in lipid use and carbon flux movement during *in vivo Mtb* growth and virulence^3^,^4^. Cholesterol plays an important role in facilitating the entry of *Mtb* into host macrophages^5^.

However, nitrogen availability and sources of nitrogen during *in vivo Mtb* growth remain a mystery. *Mtb* utilizes a variety of amino acids for its growth, including glycine and serine^6^. Studies using amino acid auxotrophs showed varying degrees of growth attenuation. *Mycobacterium bovis* Bacillus Calmette-Guerin (BCG) auxotrophic to leucine was avirulent even in immunodeficient SCID animals^7^,^8^. While, deletion of the *ilvB1* gene of branch chain amino acid metabolism in *Mtb* showed auxotrophy for the isoleucine, leucine and valine with attenuated growth in mice^9^. Also, L-arginine auxotrophy conferred on *Mtb* some of the properties of attenuated vaccine candidate^10^. These studies suggest that while some amino acids can be scavenged from the host, others need to be synthesized by *Mycobacterium* spp. This makes amino acid metabolism an attractive target for drug development.

DAO (D-amino acid oxidase: MRA_1916) is a flavoenzyme catalyzing the oxygen-dependent oxidative deamination of D-isomers of amino acids in a stereospecific manner with α-keto acids, ammonia and hydrogen peroxide being the end products. This enzymatic activity is mostly present in eukaryotic systems and in few bacteria^11^. Glycine being the simplest amino acid has no enantiomers and can act as a substrate for DAO and be converted into corresponding α-keto acid (glyoxylate) with possible entry into TCA cycle by GlcB mediated glyoxylate to malate conversion. The present study was initiated with an aim to understand the role of DAO in glycine utilization and its expression under nutritional and oxygen stress. The recombinant strains of *Mtb*-Ra with DAO and GlcB (malate synthase: MRA_1848) knockdown (DAO_KD and GlcB_KD) along with wild-type (WT) were studied on different carbon sources. Expression of DAO, GlcB, PckA (phosphoenolpyruvate carboxykinase: MRA_0219) and GlyA1 (serine hydroxymethyltransferase: MRA_1104) was studied on different carbon sources and under varying oxygen saturations.

## Results

### Cloning, expression and purification

The expression studies showed comparatively higher molecular weight of expressed proteins. The calculated molecular weight of DAO and GlcB was 34.076 and 80.40 kDa, respectively. However, expressed proteins were of ~53 kDa (DAO) (Supplementary Fig. S1a) and ~85 kDa (GlcB) (Supplementary Fig. S1c). This apparent increase in molecular weight of DAO and GlcB is due to the presence of upstream tags present in vectors used for expression of DAO and GlcB ((pET32a and pET14b respectively). The expressed proteins were present in both soluble and insoluble fractions (Supplementary Fig. S1b and S1d).

### Development of recombinant strain

**Figure 1:**
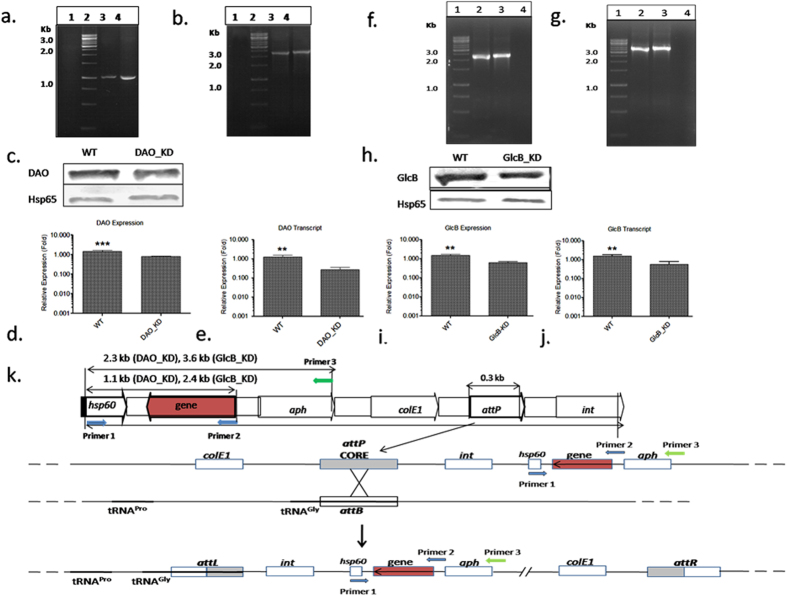
Confirmation of DAO and GlcB knockdowns: (**a**) PCR amplification using flanking region primer pair Primer 1/Primer 2, lane 1 is wild-type (WT), lane 2 is 1 kb DNA ladder (MBI Fermentas), lane 3 is pMV361-DAO plasmid construct, lane 4 is DAO_KD. (**b**) PCR using flanking region primer pair Primer 1/Primer 3: lane 1 is WT, lane 2 is 1 kb DNA ladder (MBI Fermentas), lane 3 is pMV361-DAO plasmid construct, lane 4 is DAO_KD. (**c**) Confirmation of DAO knockdown by expression analysis, WT and DAO_KD refer to wild-type and DAO knockdown strain. (**d**) Bar graph represents expression of DAO in WT and DAO_KD, Hsp65 was used as loading control, significance analysis was done by Student’s *t*-test, ***p < 0.001. (**e**) Confirmation of DAO knockdown by qRT-PCR, 16S rRNA was used as a reference for normalization. (**f**) PCR using flanking region primer pair Primer 1/Primer 2: lane 1 is 1 kb DNA ladder (MBI Fermentas), lane 2 is pMV361-GlcB plasmid construct, lane 3 is GlcB_KD, lane 4 is WT. (**g**) PCR using flanking region primer pair Primer 1/Primer 3, lane 1 1 kb DNA ladder (MBI Fermentas), lane 2 is pMV361-GlcB plasmid construct, lane 3 is GlcB_KD, lane 4 is WT. (**h**) Confirmation of GlcB knockdown by expression analysis, WT and GlcB_KD refer to wild-type and GlcB knockdown. (**i**) Bar graph represents expression of GlcB in WT and GlcB_KD, Hsp65 was used as loading control, significance analysis was done by Student’s *t*-test, ***p < 0.001. (**j**) Confirmation of GlcB knockdown by qRT-PCR, 16S rRNA was used as a reference for normalization. All immunoblots are representative of three independent experiments with similar observations. All transcript profiles are mean ± SD of three independent experiments performed in triplicates. (**k**) Diagram showing generalized knockdown construct map and its arrangement after integration into *Mtb*-Ra genome. The construction is based on the scheme proposed by Pena *et al*. (1997)^30^.

The recombinant strains were constructed by knocking down the expression of desired genes. This was achieved by gene silencing using antisense approach. For this amplicons coding for DAO and GlcB were cloned downstream of the *hsp60* promoter in an antisense orientation in mycobacterial integrative vector pMV361. The *hsp60* promoter is a constitutive promoter ensuring basal level of antisense transcripts under all conditions. The transcripts from antisense construct bind to the sense transcript being transcribed from the native gene and form a RNA duplex. This results in inhibition of native gene translation and overall decrease in cellular level of targeted gene product. Orientation of constructs in pMV361 was confirmed by PCR and pMV-DAO construct gave an amplicon of ~1.1 kb (Fig. 1a) with primer-1 and primer-2. This amplicon consisted of sequences coding for Hsp60 and DAO (Fig. 1k). Amplification with primer-1 and primer-3 flanking *hsp60* and kanamycin resistance marker (*aph*) gave an amplicon of ~2.3 kb (Fig. 1b). No amplification was seen in WT. The immunoblotting studies with anti-DAO antibody (Fig. 1c) showed down-regulation of DAO expression in DAO_KD compared to WT (Fig. 1d). This was also confirmed by transcript analysis using real-time PCR (RT-PCR) (Fig. 1e). Similarly, primer pairs (primer-1 and primer-2) flanking *hsp60* and *glcB* as well as *hsp60* and *aph* (primer-1 and primer-3) were used to confirm the GlcB antisense constructs integration in *Mtb*-Ra genome (Fig. 1f,g). The immunoblotting studies with anti-GlcB antibody (Fig. 1h) showed down-regulation of GlcB expression in GlcB_KD (Fig. 1i). This was also confirmed by transcript analysis using RT-PCR (Fig. 1j). The genotype map shows the construction scheme for recombinant development and its arrangement after integration into *Mtb*-Ra genome (Fig. 1k).

### Effect of gene knockdown on growth under carbon stress

**Figure 2:**
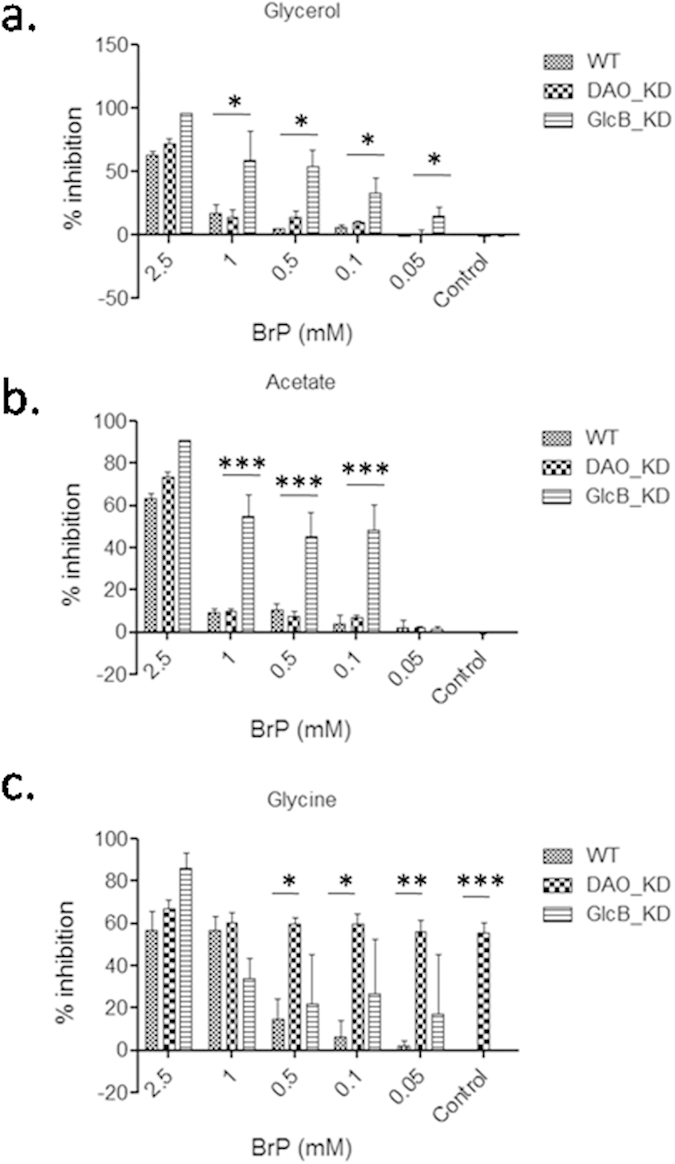
Growth inhibition in the presence of Ethyl bromopyruvate: Growth inhibition of WT, DAO_KD and GlcB_KD was studied in the presence of Ethyl bromopyruvate (BrP) on different carbon sources. (**a**–**c**) refer to growth on glycerol, acetate and glycine, respectively. The data are mean ± SD of three independent experiments performed in triplicates, significance analysis was done by Student’s *t*-test, *p < 0.05, ** < 0.01, ***p < 0.001.

The MABA assay for growth of knockdowns and WT showed inhibition of WT at 2.5 mM BrP on carbon sources studied (glycerol, acetate and glycine). A noticeable increase in inhibition of GlcB_KD compared to WT and DAO_KD was apparent while using glycerol or acetate as a carbon source (Fig. 2a,b). However, growth of DAO_KD was more inhibited on glycine containing medium compared to WT and GlcB_KD (Fig. 2c). The increased inhibition of DAO_KD on glycine as a carbon source compared to GlcB_KD and WT strains suggested poor use of glycine by DAO_KD strain. The BrP being an inhibitor of GlcB, metabolic flux through the glyoxylate shunt in recombinant strains was under dual inhibition caused by i) silencing of GlcB or DAO and ii) by BrP mediated inhibition of expressed GlcB protein leading to more growth retardation in both GlcB and DAO knockdowns.

**Figure 3:**
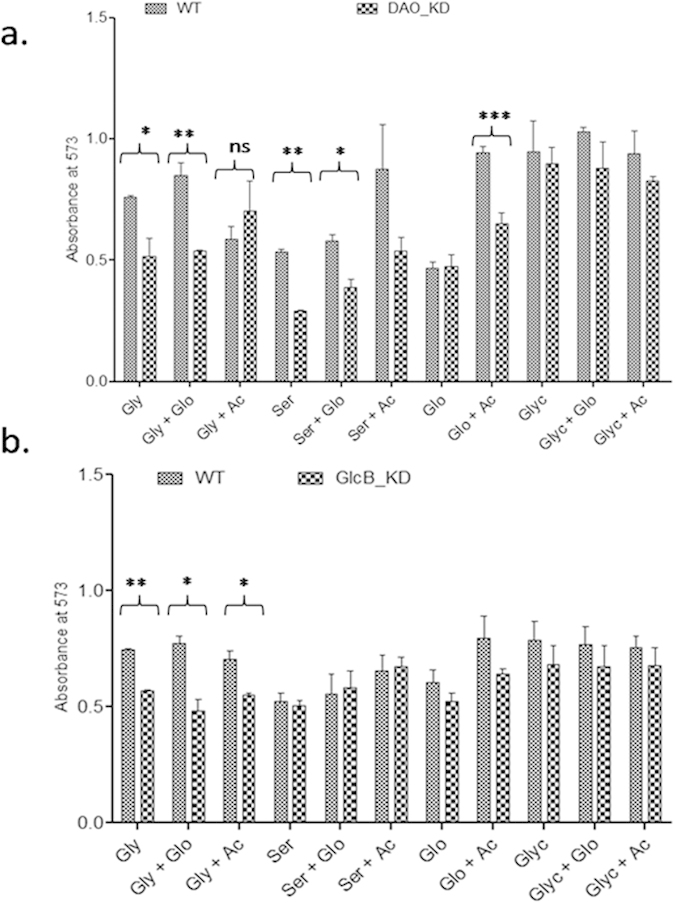
Effect of supplementation on growth: (**a**) Growth of DAO_KD and WT on different carbon sources with or without acetate or glyoxylate supplementation. (**b**) Growth of GlcB_KD and WT on different carbon sources with or without acetate or glyoxylate supplementation. Gly, Ac, Glo, Ser and Glyc refer to glycine, acetate, glyoxylate, serine and glycerol respectively. The data are mean ± SD of three independent experiments performed in triplicates, significance analysis was done by Student’s *t*-test, ^ns^*p* > 0.05, *p < 0.05, ** < 0.01, ***p < 0.001.

However, comparatively higher inhibition of DAO_KD on glycine as a carbon source required further study. Hence, we performed supplementation studies using one of these (glycerol, glycine, serine or glyoxylate) as a source of carbon with or without additional acetate or glyoxylate supplementation (5 mM) and measured the growth by MABA (Fig. 3a). The growth retardation was more appreciable in DAO_KD in comparison of WT on media containing glycine alone (*p* = 0.0449) or glycine+glyoxylate (*p* = 0.0142). Similarly, significant growth retardation was seen in DAO_KD than in WT while using serine alone (*p* = 0.0015) or serine+glyoxylate (*p* = 0.026). The growth restoration was observed upon acetate supplementation to serine containing medium. Similarly, enhanced growth was observed in glyoxylate+acetate compared to glyoxylate alone with more restoration in WT than in DAO_KD. No difference in growth of WT and DAO_KD was noticed when glycerol was used as a primary carbon source and no effect of additional supplementation of glyoxylate or acetate was noticed (Fig. 3a).

Similar growth studies undertaken with GlcB_KD showed significant growth retardation in medium containing glycine alone (*p* = 0.0017), glycine+glyoxylate (*p* = 0.0190) and glycine+acetate (*p* = 0.0244) as a carbon source. Also, no effect was noticeable on glycerol, glycerol+glyoxylate and glycerol+acetate as a carbon source (Fig. 3b).

### Localization of D-amino acid oxidase

**Figure 4:**
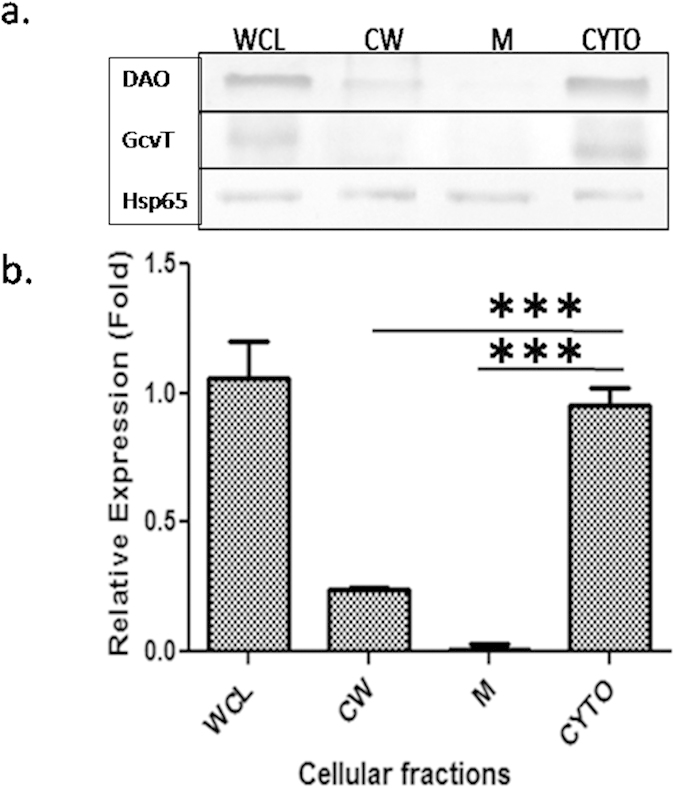
Localization of D-amino acid oxidase: WCL, CW, M and CYTO refer to whole cell lysate, cell wall, membrane and cytoplasmic fractions of WT. DAO, GcvT and Hsp65 refer to anti-DAO, anti-GcvT and anti-Hsp65 antibodies. The Hsp65 was used as a loading control, while GcvT was used as a contamination control. Immunoblot is a representative of three independent experiments with similar observations. Bar graph shows relative pixel intensities with reference to loading control. Results are mean ± SD of at least three independent experiments, significance analysis was done by Student’s *t*-test, ***p < 0.001.

The localization studies demonstrated DAO to be mostly localized in the cytoplasmic fraction followed by cell wall and negligible in the membrane fraction. Anti-GcvT antibody used as a negative control showed no contamination of respective fractions (Fig. 4a). The distribution in cytoplasmic fraction was statistically significant against membrane (*p* = 0.0004) and cell wall fractions (*p* = 0.0001) (Fig. 4b). The bioinformatic analysis of DAO showed it to be having a signal peptide as well as a hydrophobic region. This could be the reason of its detection in cell wall and membrane fractions. In the earlier mass spectrometry studies with *Mtb*, DAO was found to be present in membrane protein fraction as well as in whole cell lysate^12^.

### Effect of hypoxic stress and carbon source on gene expression

**Figure 5:**
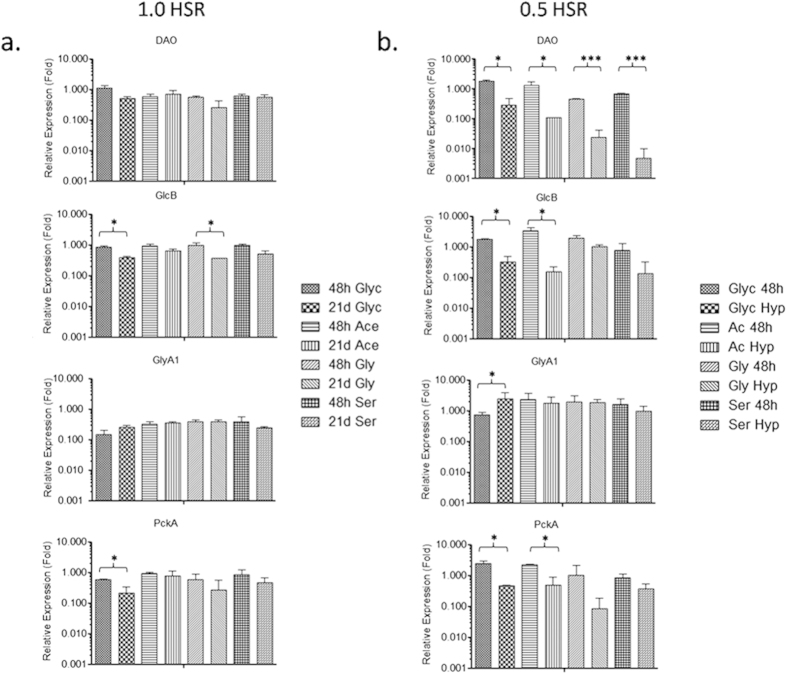
Protein expression studies under 1.0HSR and 0.5HSR: Glyc, Ac, Gly and Ser refer to glycerol, acetate, glycine and serine respectively. Bar graphs represent the normalized pixel intensities of (**a**) DAO, GlcB, GlyA1 and PckA at 48 h (48 h) and 21st day (21d) for 1.0HSR experiment. (**b**) DAO, GlcB, GlyA1 and PckA at 48 h (48 h) and under hypoxic conditions (Hyp) for 0.5HSR experiment. Hsp65 was used as a loading control in all the experiments. Results are mean ± SD of at least three independent experiments, significance analysis was done by Student’s *t*-test, *p < 0.05 and ***p < 0.001.

**Figure 6:**
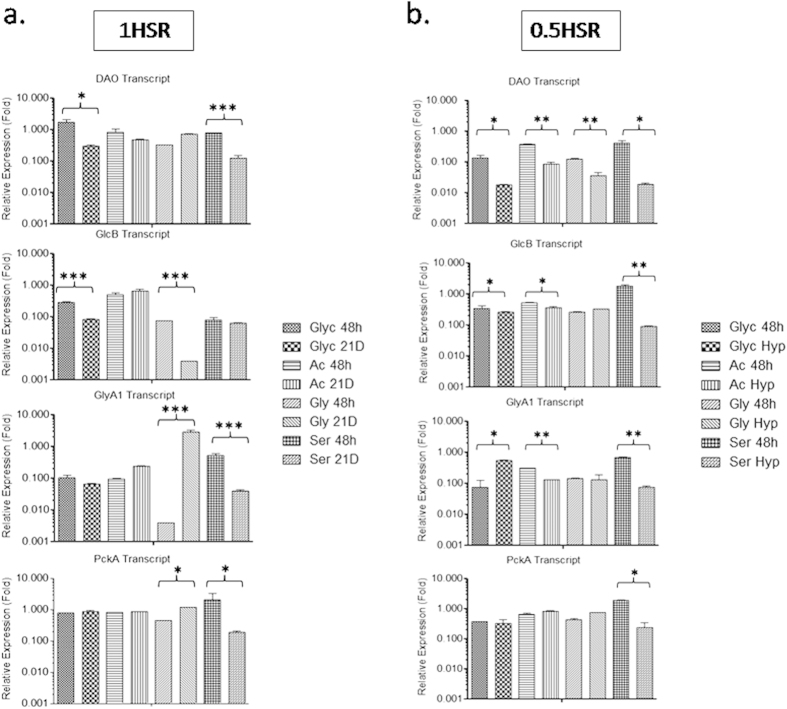
Expression studies by quantitative real-time PCR: Glyc, Ac, Gly and Ser refer to glycerol, acetate, glycine and serine respectively. Relative transcript profile of DAO, GlcB, GlyA1 and PckA in (**a**) 1HSR at 48 h (48 h) and 21^st^ day (21D), (**b**) 0.5HSR at 48 h (48 h) and after development of hypoxia (Hyp). 16S rRNA was used as a reference for normalization and relative expression folds refer to normalized levels of individual transcripts, results are mean ± SD of at least three independent experiments. Significance analysis was done by Student’s *t*-test, *p < 0.05, **p < 0.01, ***p < 0.001.

The immunoblotting and transcript profiling studies under varying oxygen saturations and nutritional conditions suggested that relative DAO expression intensities in 1HSR were comparable at 48 h and 21^st^ day on all carbon sources (Fig. 5a). Also, substantial down-regulation of DAO under hypoxic conditions in 0.5HSR on all carbon sources was observed (Figs 5b and 6b and Supplementary Fig. S2). To understand the effect of variations of DAO expression with changes in environmental conditions and its possible relationship with carbon source we studied the expression and transcript profile of GlcB, PckA and GlyA1 under partially aerobic or hypoxic conditions on glycine, glycerol, acetate and serine as a carbon source.

The differences in GlcB expression and transcript levels were substantial on glycerol and glycine as a carbon source, at 48 h and 21^st^ day under 1HSR (Figs 5a and 6a and Supplementary Fig. S2). Downregulation of GlcB expression was also noticed under 0.5HSR with considerable differences in expression and transcript levels at 48 h and under hypoxic conditions on glycerol and acetate (Figs 5b and 6b). GlcB expression and transcript levels were comparable at 48 h and under hypoxic conditions on glycine as a carbon source (Figs 5b and 6b and Supplementary Fig. S2). No difference in expression of GlyA1 was observed on all carbon sources in 1HSR (Fig. 5a) and upregulation under hypoxic conditions in 0.5HSR on glycerol as a carbon source (Fig. 5b and Supplementary Fig. S2). Differences in expression levels of PckA were observed on glycerol as a carbon source between 48 h and 21^st^ day (1HSR) as well as between 48 h and under hypoxic conditions (0.5HSR) on glycerol and acetate as a carbon source (Figs 5a,b, Supplementary Fig. S2).

**Figure 7:**
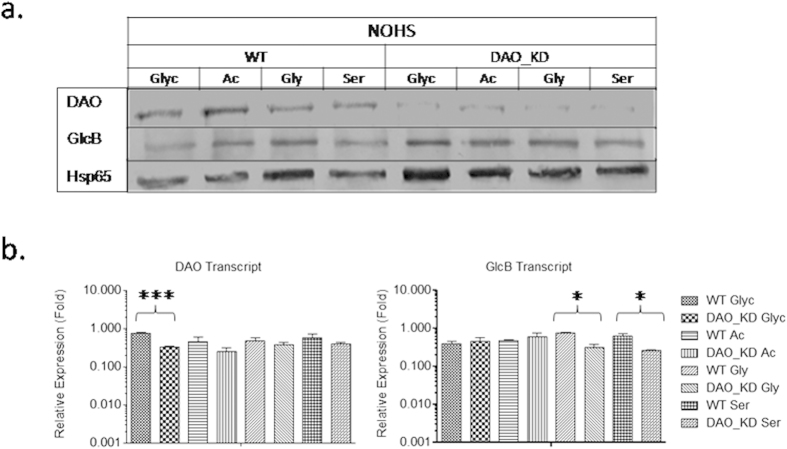
Effect of carbon source and hypoxic stress on expression of DAO and GlcB in WT and DAO_KD: Glyc, Ac, Gly and Ser refer to glycerol, acetate, glycine and serine respectively. (**a**) Immunoblot analysis of DAO and GlcB expression under NOHS, images are representative set of three independent experiments with similar observations. (**b**) Transcript profile of DAO and GlcB by quantitative real-time PCR under NOHS, 16S rRNA was used as a reference for normalization and relative expression folds refer to normalized levels of individual transcripts, results are mean ± SD of at least three independent experiments. Significance analysis was done by Student’s *t*-test, *p < 0.05, ***p < 0.001.

To further understand the effect of the carbon source, oxygen limitation and the role of growth phase on expression of DAO and GlcB, hypoxic conditions were created by minimizing the head space (NOHS). The indicator dye color loss was fast and on 5^th^ day hypoxic conditions developed. DAO expression levels and transcript folds were substantially higher in WT than in DAO_KD on glycerol as a carbon source (Fig. 7a and Supplementary Fig. S3). No difference was observed in protein expression levels of GlcB (Supplementary Fig. S3).

We have also studied the relationship between transcript and densitometry data using Pearson’s correlation coefficient analysis. Statistically significant correlation between mRNA and protein were considered as present when *r* > 0.2 and the majority of protein vs mRNA abundance data were significantly correlated (Supplementary Table S1, S2 and S3). The lack of correlation observed in a few instances could be due to various issues, including biological stability of RNA and protein, their turn-over rate, transcriptional and translational regulation of both entities. This may also be caused by the differences in sensitivities of the methods used in the study. However, the protein being the end product, the immunoblot data were taken as the final read-out. Other studies have also reported variations in concordance between protein expression vs RNA transcript levels^13^,^14^,^15^.

## Discussion

Amino acids play an important role as a source of nitrogen and also as a carbon source. Earlier studies have reported different utilization patterns among *Mycobacterium* spp. The *Mycobacterium bovis* BCG strains failed to utilize select amino acids as their sole nitrogen source for growth. This defect was explained by a lack of functional metabolic enzymes such as L-alanine dehydrogenase and in some strains due to suboptimal expression of L-serine deaminase. L-glutamine was observed to provide nutritional support comparable to that of ammonium chloride at 1 mM in *M. avium*^16^. Similarly, glyoxylate dehydrogenase activity had been observed in *Mtb*^17^ and alanine dehydrogenase *of Mtb* was found to posses both pyruvate and glyoxylate aminase activities with greater affinity to pyruvate^18^. This enzyme activity catalyzed glyoxylate to glycine conversion, but could not perform the reaction in the reverse direction. In an earlier study alanine and serine were found to be non-supportive of growth of BCG^19^; no such effect on *Mtb* growth was noticed with alanine^6^.

The serine catabolism in *Mtb* can progress through three possible routes involving serine - glycine - glyoxylate conversion mediated by serine hydroxymethyltransferase (GlyA1) and D-amino acid oxidase; through serine deaminase (SdaA) activity by converting serine to pyruvate or through serine - glycine - CO_2_ and NH_3_, using glycine cleavage system. The earlier evidence of alanine dehydrogenase (Ald)^18^ showing unidirectional glyoxylate to glycine conversion suggested possible presence of another enzyme which can perform reverse catalysis and convert glycine into glyoxylate. The end product of this enzymatic activity makes an entry into the TCA cycle through glyoxylate shunt pathway. This pathway may be an ideal recourse for a period of growth when organism is faced with limited nutrient availability. Recent studies have demonstrated that *Mtb* is capable of importing some of the amino acids from its host macrophage itself. Also, amino acids along with C1, C2 and C3 substrates were able to support growth of intracellular *Mtb*. Although, C2 carbon sources derived from acetyl-CoA/acetate were major sources of carbon, however, amino acids derived carbon was also fed to *Mtb* central metabolism^20^.

In the present study, we noticed increased inhibition of recombinants compared to WT. This suggests an increased inhibition in knockdown strains due to dual inhibitory effects of DAO/GlcB down-regulation and GlcB inhibition by BrP. Also, we observed slow growth of DAO_KD strain on glycine and serine as carbon source and growth was restored after addition of acetate. No effect of DAO or GlcB down-regulation was observed when glycerol was used as a carbon source. The glycine and glyoxylate can be interconverted by the glycine aminotransferase activity. Here, glycine can act as an amino donor and is converted into glyoxylate. Glycine can also be a substrate for DAO and be converted into glyoxylate. Both these enzyme activities can be a possible facilitator of glycine being diverted into the glyoxylate shunt pathway. Acetyl-CoA is required for subsequent conversion of glyoxylate into malate which can subsequently move into TCA and also for gluconeogenesis. The growth restoration observed in DAO_KD after acetate supplementation to glycine and serine as carbon source could be due to acetate being converted to acetyl-CoA which facilitates the assimilation through glyoxylate pathway. However, no growth difference between WT and GlcB_KD on serine and its combinations shows presence of metabolic blocks due to DAO down-regulation in DAO_KD strain. This may be leading to altered carbon utilization and results in observed growth differences. The glycine to glyoxylate conversion has been proposed for *gcvP* in *Mycobacterium smegmatis*^21^, while, alanine deaminase has been reported to also act as a glycine dehydrogenase in *Mtb*^18^.

The cellular availability of oxygen and nature of carbon source as well as availability of metabolic precursors/intermediates may be the key regulator for the shift in metabolic pathway^3^,^22^,^23^. The serine to glycine conversion by serine hydroxymethyltransferase activity and glycine to glyoxylate conversion due to D-amino acid activity, activities serves an important function of driving one carbon cycle and maintaining cellular redox balance. This cycling also caters to the synthesis of metabolic precursors. The requirement of oxygen to complete the oxidase reaction ensures that this conversion takes place primarily during aerobic conditions and to some extent under partially aerobic conditions. This may be the reason of low DAO expression under hypoxic (0.5HSR) conditions as compared with expression under aerobic (48 h) or partially hypoxic (21^st^ day in 1HSR) conditions. This expression profile is also influenced by the metabolic state of cell as was observed under induced hypoxic conditions in NOHS experiment in which stronger expression of DAO was observed compared to expression under hypoxic conditions developed in 0.5HSR. While, the hypoxia in 0.5HSR was slow to develop and cells were already in non-replicating persistence stage^24^ but hypoxia encountered by cells in the NOHS experiment happened when cells were entering log phase because of which a strong requirement of metabolic precursors led to maintaining a basal level of DAO expression in all media conditions, while a general shift down was observed under hypoxic conditions in 0.5HSR treatment.

The relative expression levels of GlcB in WT under NOHS conditions were higher than the expression levels under hypoxic conditions of 0.5HSR study. The GlcB expression under different physiological and nutritional conditions suggests that apart from acting as a metabolic enzyme, it may also have other functions^25^. The expression profile of PckA needs to be seen with respect to the cellular requirement of glycolytic intermediates and trade off with maintaining a redox balance during aerobic and hypoxic conditions leading to possible flux diversion through alternate metabolic pathways^18^,^22^,^26^. The continued expression of PckA under partially aerobic and hypoxic conditions under 1HSR and 0.5HSR could be attributed to its gluconeogenic^26^ as well as carbon fixing^20^ roles. The relatively higher concentration of accumulated CO_2_ in low head space available may lead to some of the environmental CO_2_ being fixed by PckA or being diverted to the glycolytic pathway by PckA mediated gluconeogenesis^20^. The relative expression of GlyA1 on glycine in 1HSR at 48 h and on the 21^st^ day was comparable on all carbon sources while up-regulation was observed on glycerol under hypoxic conditions. Also, relative expression levels of GlyA1 under hypoxic conditions (0.5HSR) were generally higher than those observed under partially aerobic conditions on 21^st^ day (1HSR). This differential expression of GlyA1 could be due to different head space ratios and resulting differences in oxygen saturations coupled with the cellular requirement of maintaining redox balance as well as providing metabolic precursors for maintaining the basal metabolic rates. The GlyA1 is annotated to be a serine hydroxymethyltransferase 1 with possible role in serine to glycine conversion and generation of 5, 10-methylenetetrahydrofolate which plays an important role in providing precursors for cellular redox balancing, methylation reactions and in thymidylate biosynthesis. The GlyA1 is an essential enzyme for *in vitro* growth^27^.

**Figure 8:**
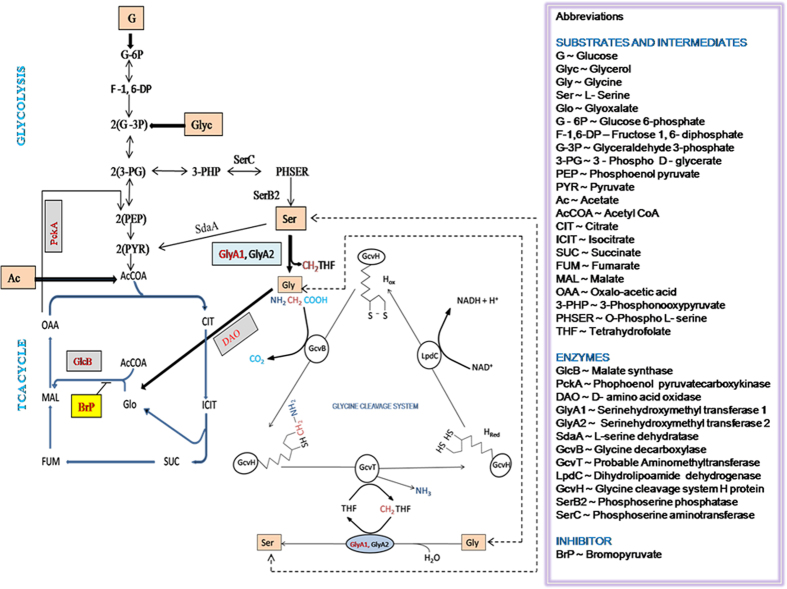
Diagrammatic representation of the proposed pathway: Pathway depicting the catalytic activities assigned to DAO, GlcB, PckA and GlyA1. BrP refers to Ethyl bromopyruvate.

The present study demonstrates that under hypoxia induced non-replicating persistence and acetate as a carbon source, DAO and GlcB expression was less compared to PckA. Also, expression of DAO was highly repressed under hypoxic conditions with glycerol, acetate, glycine and serine as a carbon source. The study also shows that the physiological state of the cell and nutritional availability influences the expression of pathway genes to maintain basal metabolism for survival under stress conditions. This cellular requirement to generate metabolic precursors and maintaining redox balance is met by cycling through metabolic pathways (Fig. 8) involving gluconeogenesis, glycolysis, amino acid metabolism, glyoxylate shunt and finally entering TCA for continuation. The possibility of flux routing through serine-glycine-glyoxylate-malate-phosphoenolpyruvate provides an opportunity for *Mtb* cells to cope with nutrient limiting conditions by cycling part of amino acids to generate metabolic precursors for carbon cycling and well as generating reducing equivalents. The metabolic networks being intricately interwoven any alteration in physiological levels of one metabolite is likely to disrupt entire homeostasis of the cell. This proposed metabolic network may be one of the many existing networks functioning in the cell to maintain its homeostasis and provide for essential reducing equivalents and metabolic precursors.

## Methods

### Cloning, expression and protein purification

Genomic DNA was isolated from *Mycobacterium tuberculosis* H37Ra (*Mtb*-Ra) growing on Middlebrook 7H9 broth (Difco) supplemented with glycerol (0.2% v/v), ADC (10% v/v) (albumin, dextrose, catalase), and Tween 80 (0.05% v/v) (MB7H9-Tween-ADC). The genomic DNA isolation was performed as per standard protocol^28^. The details are briefly described here: *Mtb* cells from 5 ml grown culture were harvested, re-suspended in 400 μl TE buffer and lysed by bead-beating (0.1 mm glass beads, 100 mg) and lysozyme treatment (30 μl lysozyme, 50 mg/ml, 1 h at 37 °C). Further, SDS (70 μl, 10%) and proteinase K (10 μl, 20 mg/ml) were added and the mixture was incubated at 65 °C for 15 min. NaCl and CTAB were added, mixed again and incubated at 65 °C for 10 min. This suspension was extracted with phenol: chloroform (1:1) followed by phenol: chloroform: isoamyl alcohol (25:24:1). The aqueous phase was precipitated with (0.6 v/v) isopropanol. Pellet was washed with ethanol (70%) and dissolved in 100 μl of TE buffer. Genomic DNA was used for PCR with an initial denaturation at 92 °C (5 min), followed by 35 cycles of 92 °C (45 sec), 58 °C (45 sec) and 72 °C (1 min). Final extension was at 72 °C for 10 min. The PCR amplicon was eluted (Sigma gel elution kit) and ligated into pGEM-T Easy vector (Promega) to give pGEM-T-*aaO* construct and transformed into *E. coli* DH5α. The clones were selected by blue/white screening. DNA mini-prep followed by restriction analysis, DNA sequencing and nucleotide blast were used for confirmation. The DAO was then mobilized into pET32a vector and transformed into *E. coli* BL21 (DE3) cells for expression studies. Similarly, *glcB* (GlcB), *glyA1* (GlyA1) and *pckA* (PckA) were cloned in pGEM-T Easy vector. Later on inserts were mobilized into pET14b (*glcB* and *glyA1*) and pET32a (*pckA*) for expression. The recombinant proteins were expressed using standard protocol and purified using Ni-NTA affinity chromatography.

### Development of recombinant strains

The inserts from pGEM-T-Easy clones were released by restriction digestion with *Eco*RI and separated on 1.2% agarose gel, eluted (Sigma gel elution kit) and ligated to *Eco*RI digested pMV361. The ligated reactions were transformed into DH5α cells and plated on LB agar plates containing kanamycin (50 μg/ml). The clones were screened for antisense orientation of inserts. Confirmed DAO and GlcB antisense constructs were individually electroporated using standard protocol^29^. The brief details are as provided: *Mtb*-Ra was sub-cultured into 100 ml MB7H9 broth and treated with glycine (1.5%) at a culture OD of 1.0–1.2 at 600 nm. The culture was harvested after 24 h incubation with glycine and pellet was washed thrice with 20 ml of 10% glycerol. Afterwards, the cells were re-suspended in 2 ml of 10% glycerol. The competent cells (400 μl) were incubated with 7 μg of antisense construct DNA at room temperature for 10 min and electroporated (0.2 cm gap cuvette) (Bio-Rad) by treating them to a single pulse of 2.5 kV with capacitance and resistance set at 36 μF and 150 Ω (ECM399, Harvard Apparatus, USA). The cells were transferred into 10 ml of MB7H9-Tween-ADC medium and incubated at 37 °C for 16 h. Cells were harvested and plated on MB7H10 agar plates supplemented with OADC and kanamycin. The plates were incubated at 37 °C for three weeks and clones were confirmed by PCR of genomic DNA. The construction of genomic map of recombinant strains with details of integration into *Mtb*-Ra genome is based on the scheme proposed by Pena *et al*. (1997)^30^.

### Development of antibodies

The animal studies were approved by CSIR-CDRI Animal Ethics Committee (approval number IAEC/2012/23N). All study methods were carried out in accordance with the approved guidelines. Antibodies were developed using the following protocol: each purified protein (250 μg) was mixed with incomplete adjuvant and injected subcutaneously to rabbit. Each booster dose comprising 150 μg of purified protein mixed with incomplete adjuvant was injected subcutaneously at 14^th^ and 28^th^ days. Afterwards, blood was collected from marginal ear vein on 35^th^ day for immunoblotting. Subsequently, 3^rd^ booster was given and afterwards booster immunizations were performed on periodic intervals. The experiment was terminated after 60 days and blood was collected. Antibodies were purified from respective serum samples using an antibody purification kit (Cell Biolabs). Purified antibodies were used for expression profiling of WT and DAO_KD under different nutritional and physiological conditions.

### Growth and inhibition studies

The MABA was used to assess the growth of WT, DAO_KD and GlcB_KD under carbon limiting conditions with either glycine or glycerol or acetate as a carbon source (1%). The basal media composition was similar to the Sauton’s media except glycerol being replaced with either glycine or acetate. 96 well plates were used for MABA assay with the final medium being 200 μl per well and different concentrations of GlcB inhibitor (BrP) were used (2.5, 1.0, 0.5, 0.1 and 0.05 mM). Control experiments were devoid of any inhibitor. The plates were sealed and incubated for 120 h, at 37 °C. Resazurin (20 μl, 0.02% w/v) was added after completion of incubation and absorbance was recorded (Multiskan Spectrum, ThermoFisher Scientific) at 573 nm after 0 and 5 h.

Another experiment with WT, DAO_KD and GlcB_KD was performed using a single carbon source (glycine/glyoxylate/serine/glycerol) along with either acetate or glyoxylate being supplemented (5 mM) as an additional carbon source. The basal media composition was similar to the Sauton’s media except glycerol was replaced with glycine, serine or glyoxalate (1%) as a carbon source. The control media used glycerol as a carbon source, other details were similar to earlier experiment with absorbance being recorded at 573 nm after 0 and 5 h.

### Subcellular localisation of D-amino acid oxidase

WT and DAO_KD log phase cultures (5 ml) were harvested, washed with PBS and re-suspended in THP solution (20 mM Tris-HCl of pH = 8.0 + 1% protease inhibitor cocktail). The cells were sonicated (Sonics, USA) for 10 min (amplitude 30%) with 10 sec pulse on/off. The subcellular fractionation was performed by following the protocol described by Giffin *et al*. (2012)^18^. Cell lysate was ultracentrifuged at 27,000 × g for 30 min at 4 °C and the supernatant (S-1) was collected. The S-1 fraction was ultracentrifuged at 100,000 × g for 2 h at 4 °C and supernatant (S-2) was collected. The pellets (P-1 and P-2) were washed with 2 ml of THP solution and were re-suspended in PBS. The protein content of P-1, P-2 and S-2 fractions representing cell wall, membrane and cytosolic components was measured by Lowry’s method and equal amount of each fraction (20 μg) was used for immunoblotting.

### Effect of carbon source and oxygen saturation on gene expression

Gene expression under nutritional and environmental stress conditions was studied by immunoblotting and quantitative real-time PCR (qRT-PCR). In immunoblotting studies, primary antibodies against DAO, GlcB, PckA and GlyA1 were used. WT strain growing on MB7H9 medium supplemented with 0.2% glycerol and 10% ADC was inoculated into four different media combinations. These media used Sauton’s medium without glycerol as the basal medium with glycerol, acetate, serine and glycine being varied as a carbon source (1%). The culture was harvested, washed and re-suspended in the same medium. The hypoxic conditions were created as per Wayne’s model^24^ and two different head space ratios (HSR) of 0.5 and 1.0 were used for study. The cells were incubated for growth and allowed to develop differential oxygen saturations due to differing head space ratios. To observe the effect of time dependent growth on gene expression and its relationship with carbon source and oxygen saturation, samples were taken at 48 h and 21^st^ day for 1HSR study and at 48 h and after complete loss of color of methylene blue for 0.5HSR study. The methylene blue was used as a hypoxia indicator and complete loss of color was treated as hypoxic condition. The harvested cells were washed with PBS and re-suspended in lysis buffer (20 mM Tris-HCl of pH = 8.0, 1.0 mM PMSF, 5.0 mM EDTA and 100 mM NaCl). The cells were lysed (Tissue Lyser, Qiagen) and the lysate was centrifuged at 13000 × g for 20 min. The supernatant was collected and its protein content was estimated by Lowry’s method. Equal amount of protein was used for blotting and Hsp65 was used as a loading control.

The effect of DAO knockdown on GlcB expression was studied using four different carbon sources (glycine, acetate, serine and glycerol @1%) under induced hypoxic conditions. Freshly grown cultures of WT and DAO_KD were harvested, washed with PBS and re-suspended in respective media. The cells in sealed tubes lacking headspace (NOHS) were incubated at 37 °C till complete loss of methylene blue color. Afterwards, cells were harvested and processed for blotting as described earlier.

RNA profiling studies were performed by isolating total RNA using TRI Reagent (Sigma). The protocol used is as described: 1.0 ml of TRI Reagent was added to the cells and vortexed vigorously for 2 min with periodic cooling in ice. Treated samples were incubated (5 min at 30 °C) and chloroform (0.2 ml/ml TRI Reagent) was added. Samples were mixed vigorously for 15 sec and incubated at 30 °C for 2 min, afterwards, centrifuged at 12,000 × *g* for 15 min at 4 °C. The upper aqueous phase was collected and precipitated with isopropyl alcohol (0.5 v/v of TRI Reagent used initially). The precipitate was washed with 75% ethanol and pelleted at 7,500 × *g* for 5 min at 4 °C. The pellet was air dried and dissolved in RNAse free water. The total RNA was estimated spectrophotometrically (Multiskan Spectrum, ThermoFisher Scientific) and 1.0 μg RNA was taken for cDNA synthesis (cDNA synthesis kit, ThermoFisher Scientific) using random hexamer primers. The cDNA was diluted (10 folds) and 1.0 μl of diluted cDNA was used for 10 μl reactions (LightCycler 480, Roche). 16S rRNA was used as a normalization control.

## Additional Information

**How to cite this article**: Singh, K. S. and Singh, S. K. The *Mycobacterium tuberculosis* H37Ra gene MRA_1916 causes growth defects upon down-regulation. *Sci. Rep*. **5**, 16131; doi: 10.1038/srep16131 (2015).

## Supplementary Material

Supplementary Information
