# Supplementary material for: The Mycobacterium tuberculosis H37Ra gene MRA_1916 causes growth defects upon down-regulation
Source: Sci Rep. 2015 Nov 4;5:16131. doi: 10.1038/srep16131 (PMC4632087; doi:10.1038/srep16131)
Supplement: Supplementary Information [file srep16131-s1.pdf]

The *Mycobacterium tuberculosis* H37Ra gene MRA_1916 causes growth defects upon down-regulation

Kumar Sachin Singh and Sudheer Kumar Singh[#]

Supplementary Figures and Tables

Supplementary Figure S1: Expression and detection of expressed proteins: (a) Expression of DAO: Lane 1 is unstained protein marker, lane 2 and 3 are uninduced and induced cultures respectively. (b) Detection of DAO by immunoblotting using anti-His antibody: lane 1 is prestained protein marker, lane 2 and lane 3 are insoluble and soluble fractions. (c) Expression of GlcB: Lane 1 is unstained protein marker, lane 2 and 3 are uninduced and induced cultures respectively. (d) Detection of GlcB by immunoblotting using anti-His antibody: lane 1 is prestained protein marker, lane 2 and lane 3 are insoluble and soluble fractions.

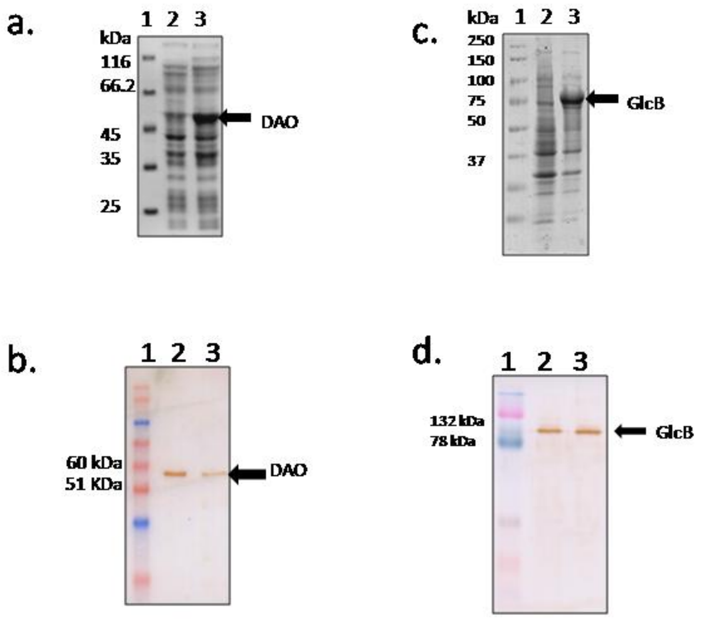

Supplementary Figure S2: Expression studies by immunoblotting: Glyc, Ac, Gly and Ser refer to glycerol, acetate, glycine and serine respectively. (a) Expression of DAO, GlcB, GlyA1 and PckA in 1HSR at 48 h (48h) and 21st day (21D). (b) Expression of DAO, GlcB, GlyA1 and PckA in 0.5HSR at 48 h and after development of hypoxia (Hyp). Hsp65 was used as a loading control in all the experiments. Results are representative of three independent experiments with similar observations.

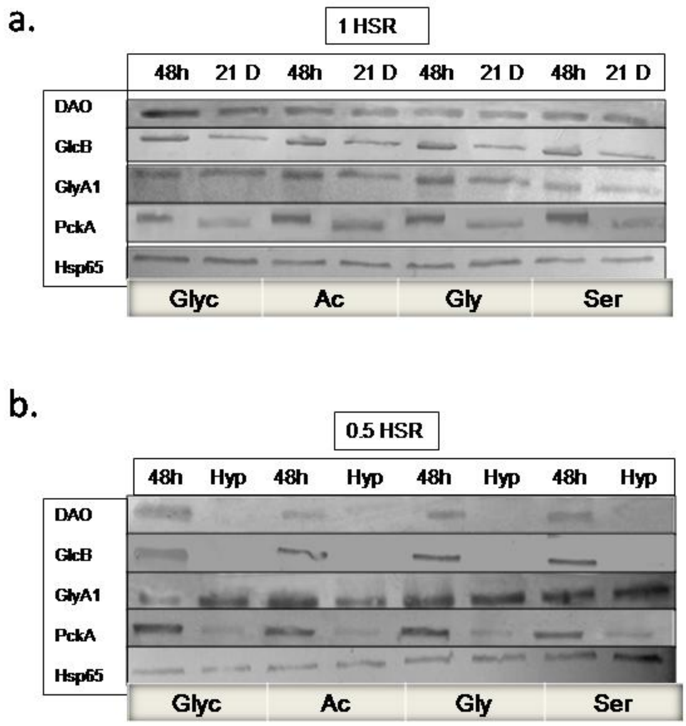

Supplementary Figure S3: Densitometry based evaluation of immunoblots in NOHS study: Glyc, Ac, Gly and Ser refer to glycerol, acetate, glycine and serine respectively. Bar graphs represent the normalized pixel intensities of (a) DAO, (b) GlcB under NOHS induced hypoxia. Hsp65 was used as a loading control in all the experiments. Results are mean ± SD of at least three indenpendent experiments, significance analysis was done by Student's *t*-test, $^{**}p<0.01$.

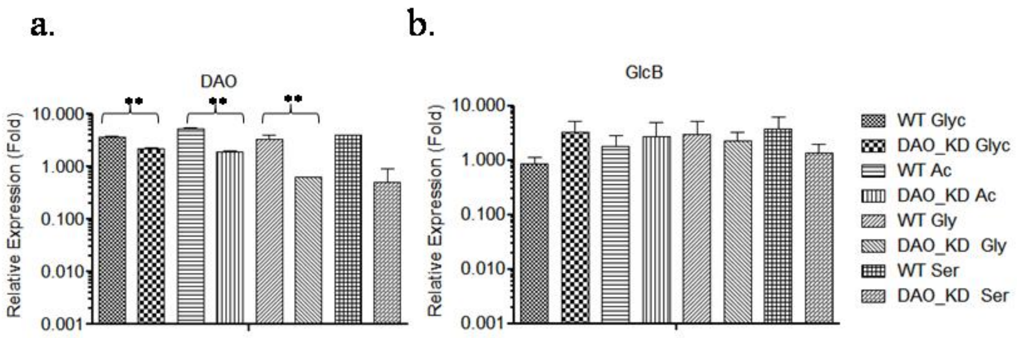

Supplementary Table S1: Correlation study between protein vs mRNA abundance under 1.0HSR treatment using Pearson's coefficient (*r*) analysis. Glyc, Ac, Gly and Ser refer to glycerol, acetate, glycine and serine as a carbon source.

mRNA vs Protein	1.0HSR			
	DAO	GlcB	GlyA1	PckA
Glyc-48h	0.853	0.989	0.898	0.905
Glyc-21D	0.946	0.992	0.829	0.88
Ac-48h	0.992	0.994	1	0.939
Ac-21D	0.308	0.999	0.853	0.998
Gly-48h	0.996	0.756	-0.024	0.732
Gly-21D	0.363	0.685	0.943	0.874
Ser-48h	0.052	0.963	0.646	0.999
Ser-21D	0.971	0.965	-0.226	0.928

Supplementary Table S2: Correlation study between protein vs mRNA abundance under 0.5HSR treatment using Pearson's coefficient (*r*) analysis. Glyc, Ac, Gly and Ser refer to glycerol, acetate, glycine and serine as a carbon source.

mRNA vs Protein	0.5HSR			
	DAO	GlcB	GlyA1	PckA
Glyc-48h	0.929	0.721	0.998	0.745
Glyc-hyp	0.964	0.946	0.965	0.955
Ac-48h	0.992	0.973	0.939	0.983
Ac-hyp	0.993	0.863	0.759	0.946
Gly-48h	0.93	0.976	0.993	0.565
Gly-hyp	0.866	0.997	0.855	0.349
Ser-48h	0.924	0.831	0.903	0.964
Ser-hyp	0.991	0.978	0.943	0.831

Supplementary Table S3: Correlation study between protein vs mRNA abundance under NOHS treatment using Pearson's coefficient (*r*) analysis. Glyc, Ac, Gly and Ser refer to glycerol, acetate, glycine and serine as a carbon source.

mRNA vs Protein	NOHS	
	DAO	GlcB
WT Glyc	0.729	0.737
DAO_KD Glyc	0.379	0.999
WT-Ac	0.995	0.574
DAO_KD Ac	0.913	0.951
WT Gly	0.147	0.433
DAO_KD Gly	0.937	-0.476
WT Ser	0.919	-0.046
DAO_KD Ser	0.416	0.667
